# Relationship among serum levels of IL-6, sIL-6R, s gp130 and CD126 on T-cell in HIV-1 infected and uninfected men participating in the Los Angeles Multi-Center AIDS Cohort Study

**DOI:** 10.1371/journal.pone.0290702

**Published:** 2023-10-09

**Authors:** Najib Aziz, Roger Shih, Nicole Alexopoulos, Beth D. Jamieson, Matthew J. Mimiaga, Otoniel Martinez-Maza, Roger Detels

**Affiliations:** 1 Department of Epidemiology, Fielding School of Public Health, University California Los Angeles, Los Angeles, California, United States of America; 2 Department of Medicine, David Geffen School of Medicine, University California Los Angeles, Los Angeles, California, United States of America; 3 Department of Psychiatry & Biobehavioral Sciences, David Geffen School of Medicine, University California Los Angeles, Los Angeles, California, United States of America; 4 Departments of Obstetrics & Gynecology and Microbiology Immunology and Molecular Genetics, UCLA, David Geffen School of Medicine, Los Angeles, California, United States of America; University of Verona, ITALY

## Abstract

**Introduction:**

Interleukin 6 (IL-6) activates cells through its unique heterodimeric signaling complex of IL-6 receptor (IL6R) subunit and interleukin 6 signal transducer β-subunit glycoprotein 130 (gp130). The objective of this study was to investigate associations among serum levels of IL-6, sIL-6R, sgp130 and relative fluorescence intensity (RFI) of the α-subunit of the IL-6 receptor (CD126) on T-cells of HIV-1 infected and uninfected men.

**Methods:**

Blood samples were obtained from 69 HIV-1-infected men on Highly Active Antiretroviral Therapy (HAART) with mean age of 49.1 and 52 HIV-1-uninfected with mean age of 54.3 years -. All men were participating in the Los Angeles Multi-Center AIDS Cohort Study (MACS). Serum levels of IL-6, sIL-6R, sgp130 were measured by enzyme-linked immunoassays and T-cell phenotypic analysis and RFI of CD126 on CD4^+^ and CD8^+^ by flow cytometry.

**Results:**

Mean serum levels of IL-6, sIL6R, sgp130 and of CD126 RFI on CD4^+^ were 4.34 pg/mL, 39.3 ng/mL, 349 ng/mL and 526 RFI respectively for HIV-1-infected men and 2.74 pg/mL, 41.9 ng/mL, 318 ng/mL and 561 RFI respectively for HIV-1-uninfected men. The mean serum concentrations of IL-6, sIL-6R in HIV-1-infected and uninfected men were not significantly different (p>0.05). There was a positive correlation between plasma HIV-1 RNA and the levels of IL-6 (p<0.001), sIL6R (p = 0.002) but no correlation with sgp130 (p = 0.339). In addition, there was a negative correlation between serum levels of IL-6 with RFI of CD126 on CD4^+^ (p = 0.037) and a positive correlation between serum levels of sgp130 (p = 0.021) and sIL-6R in HIV-1-infected men.

**Conclusion:**

Knowledge of biological variation, differences in the blood levels of biomarkers among healthy individuals and individuals experiencing illness, are very important for selection of appropriate tests for stage and progression of disease. Our data suggest no correlation among IL-6, and sIL-R6, in the treated phase of HIV-1 infection. The action and blood level of IL-6 and its receptors may be different at each stage of a disease progression.

## Introduction

Interleukin 6 (IL-6) is a multifunctional cytokine with both pro- and anti-inflammatory functions. Picogram per milliliter (pg/mL) of IL-6 can be measured in the blood of healthy individuals. IL-6 expression is upregulated in nearly all pathophysiological states [1]. Human interleukin-6 (IL-6) is secreted by T-cells, B-cells, monocytes, macrophages, fibroblasts, keratinocytes, endothelial cells, mesangial cells, adipocytes and some tumor cells. It regulates the immune response, hematopoiesis, synthesis of acute phase proteins and inflammation [2].

Interleukin 6, a small secreted glycoprotein cytokine, activates cells through its unique heterodimeric signaling complex. The complex consists of two molecules: an IL-6 receptor (IL6R) α-subunit or CD126 and interleukin 6 signal transducer ß-subunit glycoprotein 130 (gp130) or CD130 [1]. The gp130 subunit also is common signal transducer for several other cytokines such as; IL-11, IL-27, and Leukemia inhibitory factor [3].

Interleukin 6 receptor (CD126) is present on only a few cell types within the body, such as hepatocytes and some leukocytes, whereas gp130 is expressed on all cells. Since all cells in the body express gp130 then theoretically all cells can be activated by the IL-6/sIL-6R complex [4, 5]. Nanogram per milliliter (ng/mL) of sIL-6R also can be measured in the serum, plasma, urine, cerebral spinal fluid (CSF), and synovial fluid (SF) [6].

Receptors of each cytokine exist in two forms: cell membrane-bound and soluble form in the circulating blood or body fluids. Most soluble receptors are antagonists which compete with their membrane counterpart for their ligands. Some of the receptors such as sIL-6R are agonists. In this case IL-6 and sIL-6R make a complex of IL-6 and sIL-R6 and bind to a second subunit of gp130 on the surface of a target cell. The complex then initiates intracellular signal transduction and the cell responds to IL-6. In vivo the IL-6/sIL-6R complex stimulates several types of target cells which lack the membrane-bound IL-6R, a process called trans-signaling [7, 8]. The soluble form of gp130 (sgp130) has an antagonistic effect on IL-6/sIL-6R and blocks the action of IL-6 on target cells.

Basically, the combination of sIL-6R and sgp130 in serum form a buffer system that neutralizes the excess levels of blood IL-6 in healthy individuals. Anything that unbalances this buffer system can affect the pathophysiology of a disease [9].

Interleukin 6 signaling through the membrane bound IL-6R (classic signaling) leads to activation of the immune system through anti-inflammatory pathways (STAT-3) which causes intestinal epithelial proliferation, inhibition of epithelial cell apoptosis, induction of the hepatic acute phase response, acute phase protein secretion, fever induction and B-cell differentiation into plasma cells to produce antibodies [1].

Interleukin 6 signaling by the sIL-6R (**trans-signaling**) promotes pro-inflammatory pathways by inhibition of lamina propria T-cell apoptosis, malignant proliferation of epithelial cells, recruitment of inflammatory cells (mononuclear) and maintenance of TH_**17**_ phenotype in inflamed tissues. Trans-signaling appears to be only activated during chronic inflammation and cancer [8] and it is responsible for the pro-inflammatory effects of IL-6 in the absence of IL-6R. In addition, IL-6 does not appear to bind to gp130 [5].

Expression of CD126 or IL-6R on a subset of human T-cells has previously been investigated and confirmed that CD126 is expressed on the majority of CD4^+^ T-cells, but is expressed on a smaller population of CD8+ T-cells [10–12].

Individuals in the early phase of their HIV-1 infection show elevated plasma IL-6 and sIL-6R levels which suggests that HIV-1 plays a central role in inducing and increasing production of IL-6 and sIL-6R in vivo. In addition, phytohemagglutinin (PHA) stimulated peripheral blood mononuclear cells (PBMC) of HIV-1-uninfected individuals can significantly release sIL-6R compared to the non-stimulated PBMC [13, 14].

HIV-1 infection is associated with chronic inflammation and activation of immune cells through trans-signaling pathways. Immune activation and inflammation are associated with high plasma levels of IL-6 and sIL-6R, not only during untreated HIV-1 infection, but during other chronic illness such as multiple sclerosis [15], B-cell lymphomas [16], and Type 2 Diabetes [9].

Interleukin 6 and its receptors play an important role in pathogenesis of diseases including HIV-1 infection. Increased serum levels of sIL-6R and IL-6 have been observed during the chronic stage of untreated HIV-1-infection, most likely due to residual ongoing production of virus or viral particles [17, 18]. It would be reasonable to postulate that interrupted production of sIL-6R and sgp130, may play a critical role in the development and regression of AIDS- Kaposi’s sarcoma (KS) [19].

The correlation among IL-6 and sIL-6R and especially sgp130 receptors is not clearly documented for people living with HIV-1 (PLWH). Therefore, we proposed to examine blood levels of IL-6, sIL-6R, sgp130 and RFI molecular expression of CD126 (IL-6R) on CD4^+^ T-cells, and CD8^+^ T-cells in the blood. The specific aim of this study was to evaluate the strength of the correlations among these laboratory biomarkers in HIV-1-infected men on HAART.

## Material and methods

### Study participants

This retrospective and cross-sectional study investigated the serum levels of IL-6, sIL-6R, sgp130 and RFI expression of CD126 on CD4^+^ and CD8^+^ T-cells in 121 HIV-1-infected and uninfected men who have sex with men (MSM) of multiple races ranging between 19–85 years-old at the time they provided blood specimens.

Stratified random sampling was done based on the HIV-1 serostatus, age of participants, and the order the blood samples arrived in the laboratory. Ultimately, 69 HIV-1-infected on HAART and 52 HIV-1-uninfected men from a total of 653 men enrolled in the UCLA site of Multi-center AIDS Cohort Study (MACS) were chosen to be studied. The MACS was initiated in 1983 to conduct cohort studies of men who have sex with men, and investigate the natural history of acquired immunodeficiency syndrome (AIDS) [20].

The University California Los Angeles (UCLA) institutional review board (IRB) for human subjects approved the protocol (IRB#10–0011677) and this study was conducted in adherence to the Declaration of Helsinki. Blood samples from the participants were obtained biannually per written informed consent and deposited in both local and a single national repository for use with approved sub-studies.

Blood was collected into 10 mL plastic serum separator tube (SST) for obtaining serum and into two 5 mL plastic lavender top tubes containing anticoagulant Ethylenediaminetetraacetic Acid (EDTA), (Becton Dickinson VACUTAINER Systems, New Jersey) for complete white blood cell (WBC) count, and differentiations, as well as cells surface phenotyping by flow cytometer. Serum and plasma were separated by centrifugation from the plastic SST and lavender top tubes. Aliquots of serum and plasma were stored at -70° C for batch tests of HIV-1 RNA quantification and biomarkers

Lymphocyte Immunophenotyping was performed in real time in-house using a Becton Dickenson (BD) FacsCalibur flow cytometer, and white blood cell (WBC) count and differentiations were performed by Quest Diagnostics Laboratories.

### Laboratory assays

#### IL-6 assay

Serum IL-6 concentrations were measured using a highly sensitive sandwich enzyme immunoassay from R&D Systems (Minneapolis, MN, USA). The lower limit of detection was 0.039 pg/mL and the intra-assay coefficient of variation (CV) was determined to be 6.9% and 7.4% for assay control samples with mean concentrations of 0.436 pg/mL (*n* = 20) and 5.53 pg/mL (*n* = 20), respectively.

#### sIL-6R assay

Serum sIL-6R concentrations were measured using a sandwich enzyme immunoassay from R&D Systems (Minneapolis, MN, USA). The lower limit of detection was 6.5 pg/mL and the intra-assay coefficient of variation (CV) was determined to be 8.6% and 2.3% for assay control samples with mean concentrations of 134 pg/mL (*n* = 20) and 1669 pg/mL (*n* = 20), respectively.

#### sgp130 assay

sgp130 concentrations were measured using a sandwich enzyme immunoassay from R&D Systems (Minneapolis, MN, USA). The lower limit of detection was 0.05 ng/mL and the intra-assay coefficient of variation (CV) was determined to be 4.3% and 4.7% for assay control samples with mean concentrations of 0.70 ng/mL (*n* = 20) and 8.43 ng/mL (*n* = 20), respectively.

### Lymphocyte immunophenotyping

The tests was performed by the Los Angeles Clinical Immunology Research (CIRL) flow cytometry laboratory which participated in the Quality Assurance (QA) of the College of American Pathologist (CAP) program, using the BD FACSCalibur Flow Cytometry System. Flow cytometry data were analyzed by utilizing Cellquest^R^ software of Becton Dickinson Immunocytometry Systems (BDIS).

### Staining methods

EDTA anticoagulated blood was collected by venipuncture and held at room temperature until staining, which was performed within 24 hours of collection. The Lyse No Wash (LNW) method was performed for Tritest (Tubes #1 and #2) and a lyse with wash method was done for tubes #3 and #4. 50 μL of undiluted whole blood was added into each 12 x 75mm tubes containing 20 μL of undiluted BD Tritest CD3FITC/CD4PE/CD45 PerCP (Tube #1) and BD Tritest CD3FITC/CD8PE/CD45PerCP antibodies (Tube #2), CD3FITC/CD126PE/CD4PerCP (Tube #3) and CD3FITC/CD126PE/CD8 PerCP (Tube #4) (DB Biosciences). The tubes were vortexed gently, and then incubated for 15 minutes at room temperature in the dark. After the incubation, 450 μL 1x BD FACSLyse lysing solution was added into all 4 tubes and followed by another vortex and 15 minutes incubation. Tube #3 and tube #4 after lysing were centrifuged at 800 RPM for 5 minutes, the supernatant of tubes #3 and #4 was aspirated without disturbing the cells pellet aspirated and 0.5 mL washing buffer (2% FBS) was added into the tubes which were again centrifuged at 800 RPM for 5 minutes. The supernatant was aspirated and 0.5 mL washing buffer was added into those tubes, vortexed and then analyzed.

The mean measurement of Relative Fluorescence Intensity (RFI) of CD38 and HLA-DR on CD8^+^ and CD126 on CD4^+^ and CD8^+^ were calculated from the mean channel numbers using the calibration procedure [21, 22]. The data from each tube acquired by FACSCalibur flow cytometer and lymphocyte immunophenotyping of flow cytometry data were analyzed by using Cellquest software (BDIS).

#### HIV-1 RNA quantification by Reverse Transcription-Polymerase Chain Reaction (RT-PCR)

20 vials of 1 mL each of frozen EDTA plasma were batched and shipped on dry ice by overnight shipment to the TriCore Reference Laboratories (Albuquerque, NM). Plasma HIV-1 RNA was quantified by amplification of nucleic acid using a COBAS TaqMan Analyzer.

### Statistical analysis

A four-parameter curve-fitting program was used to generate calibration curves and computation of the unknown concentration of each ELISA biomarker. Descriptive statistics were used to illustrate the levels of each of the blood markers among the study groups. The statistical significance difference between means of biomarkers among HIV-1-infected and uninfected was analyzed by using a Student’s t-test with Satterthwaite approximation to account for unknown sample variances [23].

In results with p<0.05, the difference in the mean values of the two groups is greater than would be expected by chance; in this case, there is a statistically significant difference between two groups. In the results with p>0.05, the difference in the mean values of the two groups is not discrepant enough to reject the possibility of random sampling variability and is not statistically significant different between the two groups. Also, the nonparametric Wilcoxon Rank Sum Test was used for comparing the median differences between serum levels of IL-6, sIL-6R, and sgp130 from HIV-1-infected and uninfected men.

Pearson and Spearman correlation coefficient analyses were used to analyze the association between blood biomarkers. The correlation coefficient, r, ranges from -1 to +1. The pair (s) of variables with positive correlation coefficient and p<0.05 tend to increase together. For pair (s) of variables with negative correlation coefficient and p<0.05, one variable tends to decrease while the other increases. For p>0.05 there is no significant correlation between the two biomarkers. Data were analyzed using SAS version 9.4 (SAS Institute, 2013). Graphs were produced by using SigmaPlot software version 14 (Jandel Scientific, San Rafael, CA 2008).

## Results

### A. Demographic and laboratory data for HIV-1-infected and uninfected men

The HIV-1-infected group were in the chronic stage of infection and on HAART. This group consisted of 69 men and included 14 American Indian/Alaskan Native, 18 African American (1 with Chronic Kidney Disease (CKD)), 4 multi-racial (1 with AIDS), and 33 white Caucasians (2 with AIDS, 3 experiencing CKD, 2 with squamous cell carcinoma of the anus) with mean and median age of 49.1 and 51 years old respectively. At the time of selection for this study, the CD4 counts for three cases of previously diagnosed AIDS were 847 cells/μL, 288 cells/μL, and 204 cells/μL. Out of 69 HIV-1-infected men, 17 were current smokers, 35 were former smokers, 15 never smoked, and two were missing smoking status reports.

The HIV-1-uninfected group consisted of 52 HIV-1-uninfected men who have sex with men (MSM). They were all documented to be HIV-1 seronegative at the time of this study and included 6 African American, 5 multi-racial (1 with chronic liver disease), and 41 white Caucasians (2 with CKD, 1 individual with Chronic Obstructive Pulmonary Disease (COPD), 1 with Parkinson’s, and 1 recorded acute kidney injury) with mean and median age of 54.3 and 51.0 years old. Of the 52 HIV-1-uninfected men, 11 were current smokers, 25 former smokers, and 17 never smoked. The mean, median, range data of age, WBC, lymphocytes, T-cell phenotype, and serum levels of IL-6, sIL6-R, sgp130 of HIV-1-infected and uninfected men are presented in Table 1.

**Table 1:** Demographic and laboratory data of HIV-1-infected and HIV-1-uninfected men.

Markers/unit	HIV-1-uninfected men (n = 52)			HIV-1-infected men (n = 69)		
	Mean	Median	Range	Mean	Median	Range
**Age (year)**	54.3	55.5	24–81	49.1	51.0	24–73
^a^ **WBC 10^3^ /μL**	6.8	6.4	3.8–14.2	5.8	5.9	3.1–10.5
**Lymphocyte (%)**	32.4	32.0	20–48	35.8	36.0	16–58
^b^ **CD4^+^T-cell/ μL**	959	900	480–1602	614	587	15–1790
^b^ **CD8^+^T-cell/ μL**	621	590	212–1316	882	814	263–1984
^c^ **RFI of CD126 on CD4^+^T-cell**	561	567	157–995	526	604	56–1129
**RFI of CD126 on CD8^+^T-cell**	138	136	56–341	118	106	34–289
**RFI of CD38 on CD8^+^T-cell**	372	274	57–1276	1042	529	62–6012
**Serum IL-6 (pg/mL)**	2.74	1.72	0.41–12.7	4.34	1.68	0.64–65.18
**Serum sIL-6R(ng/mL)**	41.9	41.6	21.1–72.4	39.3	34.8	20.4–126
**Serum sgp130 (ng/mL)**	318	320	223–608	349	323	196–641
**HIV-1 viral RNA (copies)**	-----	-----	------	34428	40	1–10^6^

^a^ White Blood cell,

^b^ Absolute count (Abs),

^C^ relative fluorescence intensity

### B. Comparison of blood biomarkers

The difference between serum levels of IL-6, sIL-6R, sgp130 and T-cell phenotype of Abs CD4^+^, Abs CD8^+^, RFI expression of CD126 on CD4^+^, on CD8^+^, and RFI expression of CD38 on CD8^+^ of HIV-1-infected and uninfected men were calculated using the unpaired t-test. The differences in the mean value of sgp130, Abs count of CD4^+^, Abs count of CD8^+^, and RFI expression of CD38 on CD8^+^ were statistically significant between HIV-1-infected and uninfected men. However, the differences in the mean values of IL-6, sIL-6R, RFI of CD126 on CD4^+^ and on CD8^+^ were not significantly different among HIV-1-infected and uninfected men. Also, no significant differences were seen in the median value the data of HIV-1-infected and uninfected men for IL-6, sIL-6R, and sgp130 by Wilcoxon Rank Sum test with p values of 0.9603, 0.0808, and 0.1495 respectively.

The mean values of IL-6 and sgp130 in HIV-1-uninfected men were lower compared to HIV-1-infected men, but the mean value of sIL-6R in HIV-1-uninfected men was higher compared to HIV-1-infected men. In addition, there was no statistically significant difference in the mean values of serum levels of IL-6 and sIL-6R of the HIV-1-infected and uninfected men (p = 0.158 and p = 0.335 respectively), but there was a statistically significant difference in the mean value of serum levels of sgp130 (p = 0.040) among HIV-1-infected and uninfected men.

**Fig 1:**
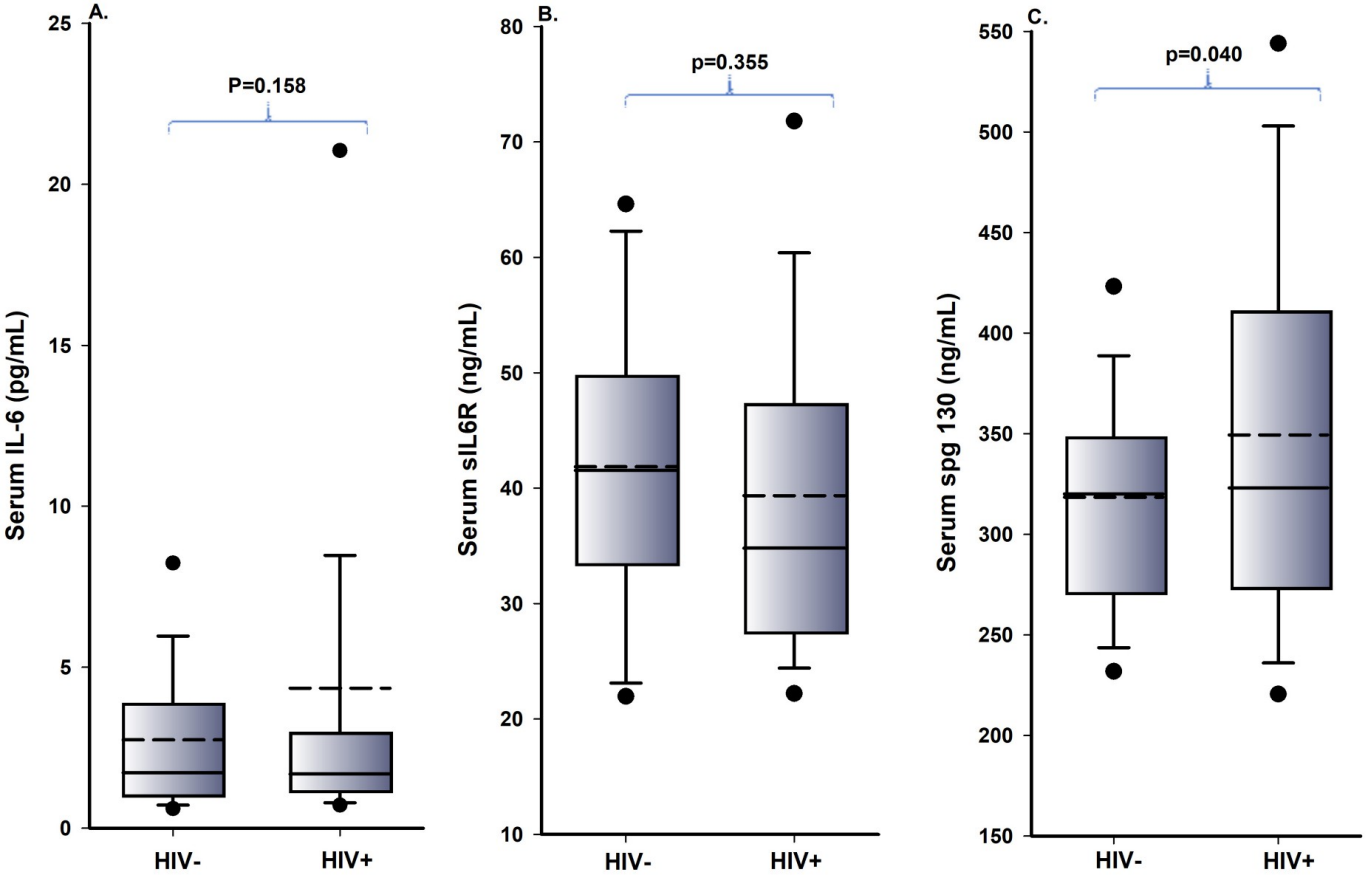
**(A-C).** Boxplot levels of serum IL-6, serum sIL-6R and serum sgp130 of HIV-1-infected and uninfected men. The solid lines represent the median, the box represents the 25th to 75th percentiles and the dashed line corresponds to the mean. The lower and upper horizontal bars represent the 5th and 95th percentiles, respectively.

**Fig 2:**
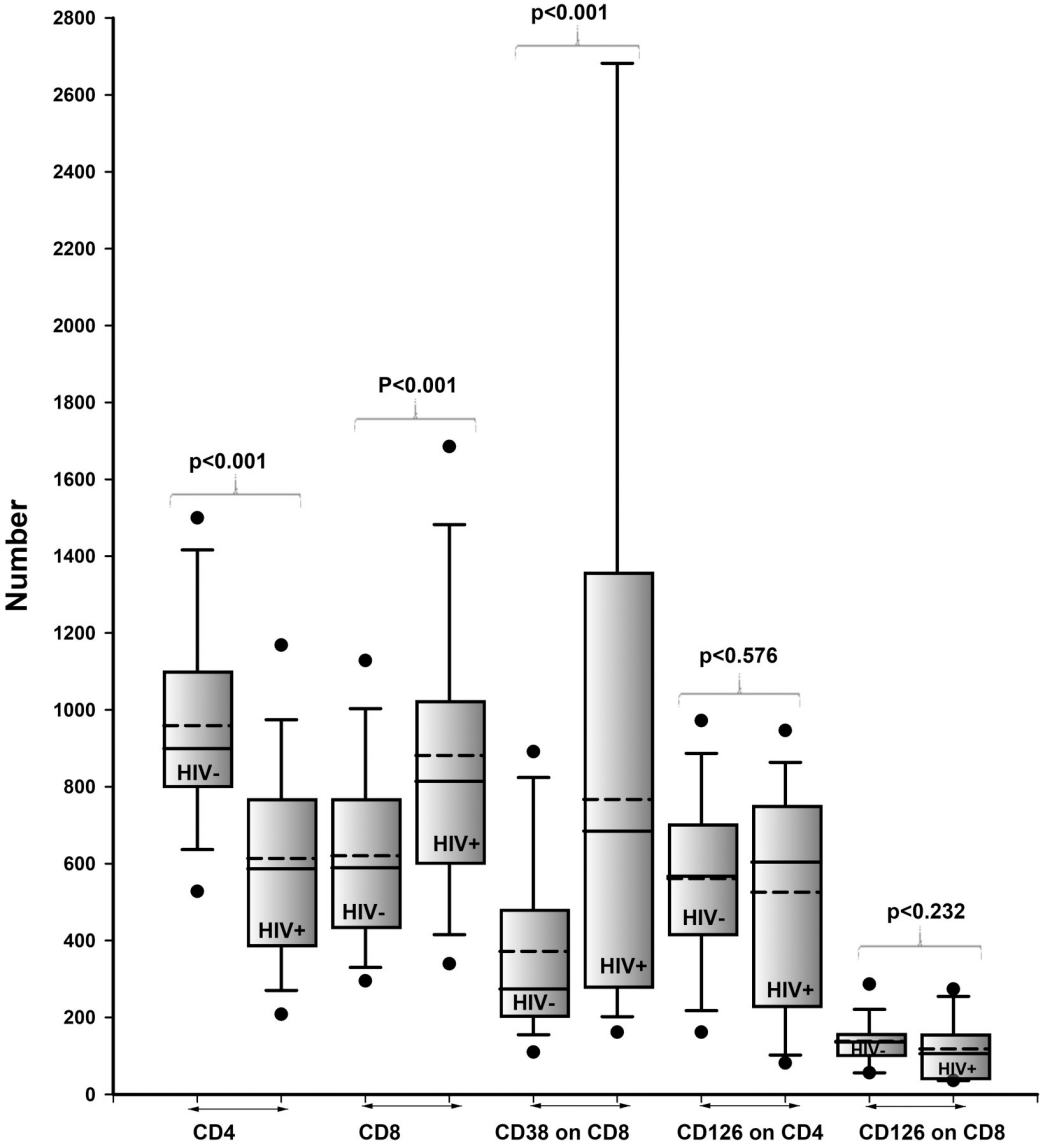
Boxplot levels of Abs CD4^+^ T-cell, Abs CD8^+^ T-cell, *RFI of CD38 on CD8^+^, RFI of CD126 on CD4^+^ and RFI of CD126 on CD8^+^ of HIV-1-infected and uninfected men. The solid lines represent the median, the box represents the 25th to 75th percentiles and the dashed line corresponds to the mean. The lower and upper horizontal bars represent the 5th and 95th percentiles, respectively. The Y axis number represent CD4^+^, CD8^+^ absolute count/**μ**L, RFI of CD38 on CD8^+^, and RFI of CD126 on CD4^+^ and CD8^+^.

The mean, median and p values between the HIV-1-infected and uninfected men are presented as boxplots in Figs 1 and 2.

### C. Relationship between blood biomarkers

The data showed that there were no significant correlations among serum levels of IL-6, sIL-6R and sgp130 in HIV-1-infected and uninfected men (except between IL-6 and sgp130, IL-6 and CD126 RFI on CD4^+^) Sl and S2 Tables. In addition, there was no significant correlation among IL-6 and sIL-6R and sgp130 when we divided HIV-1-infected men based on the serum levels of IL-6 into two groups of IL-6 ≤1.80 pg/mL (N = 38) and IL-6>1.80 pg/mL (N = 31) S5 and S6 Tables.

**Fig 3:**
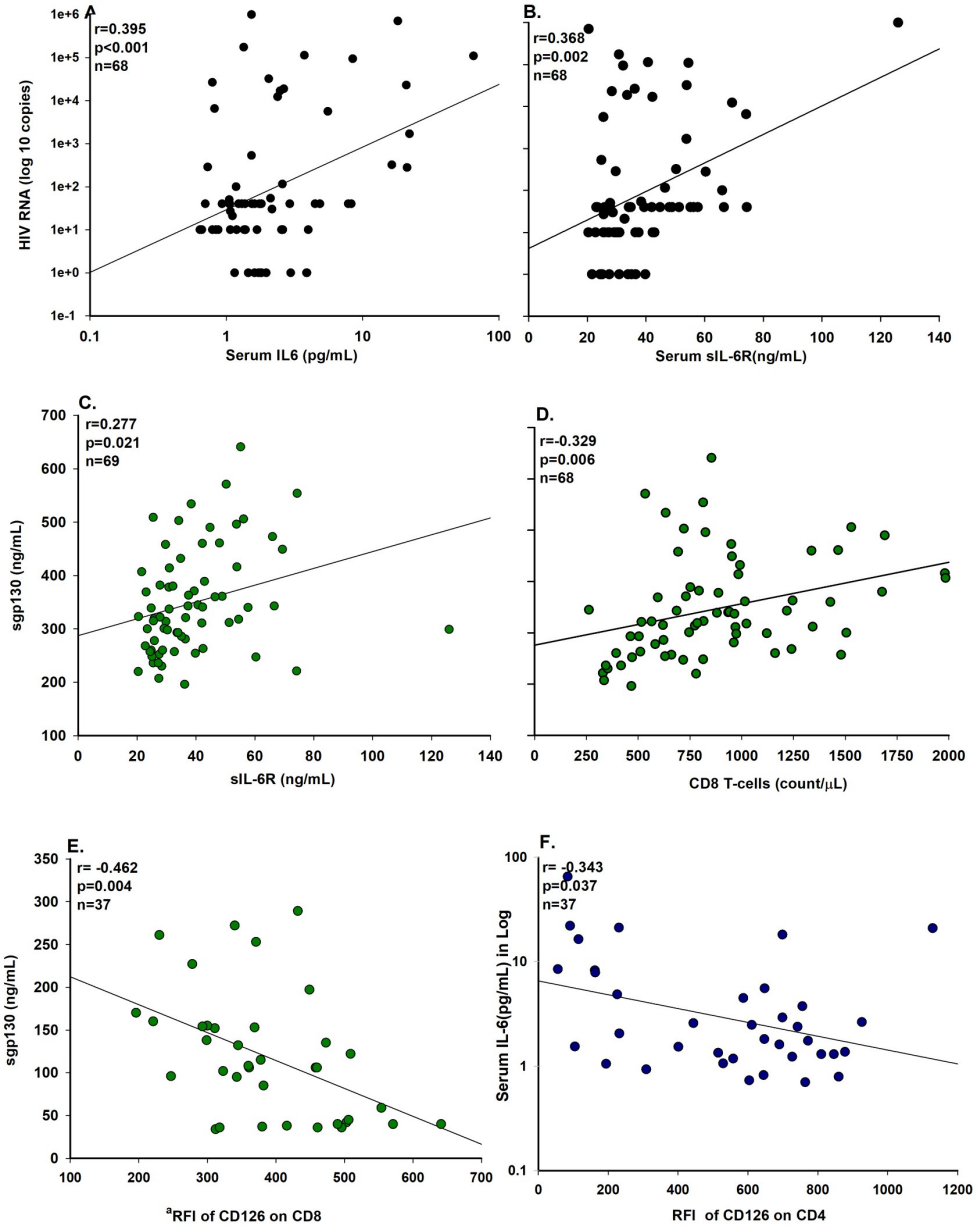
Correlation coefficients biomarkers of HIV-1-infected men. Correlation coefficients of HIV-1 RNA, with serum levels of IL-6 (A), and sIL-6R (B), correlation coefficients of serum levels of sgp130 and with sIL-6R (C), with CD8 count (D) and with ^a^RFI of CD126 on CD8 T-cell (E), and correlation coefficients of serum level of IL-6 with RFI of CD126 on CD4 (F). ^a^RFI; relative fluorescence intensity.

The correlation coefficients for some of the biomarker detected in HIV-1-infected individuals are plotted in Fig 3A–3F.

There were positive correlations between blood levels of HIV-1 RNA and IL-6 (r = 0.395, p<0.001) Fig 3A, sIL-6R (r = 0.368, p = 0.002) Fig 3B, and between sgp130 and sIL-6R (r = 0.277, p = 0.021) Fig 3C and sgp130 and abs CD8^+^ (r = 0.329, p = 0.006) Fig 3D. Also, there were negative correlations among serum sgp130 and RFI of CD126 on CD8^+^ (r = -0.462, p = 0.040) Fig 3E, and of serum IL-6 among RFI of CD126 on CD4^+^ (r = -0.343, p = 0.037) Fig 3F and the IL-6 outlier value of 65.18 pg/mL excluded for plot of the Fig 3F.

There was a strong positive correlation among RFI of CD126 on CD4^+^ and RFI of CD126 on CD8^+^ (r = 0.774, p<0.001) S1 Table. In addition, there was no significant correlation among IL-6 and sIL-6R and sgp130 (with exception of sIL-6R with sgp130 for IL-6>1.8 pg/mL) when we divided HIV-1-infected men based on the serum levels of IL-6 into two groups of IL-6 ≤1.80 pg/mL (N = 38) and IL-6>1.80 pg/mL (N = 31) S5 and S6 Tables.

There were correlations among IL-6 and RFI of CD38 on CD8^+^, WBC, lymphocytes and other biomarkers in HIV-1-uninfected men S2 and S4 Tables.

## Discussion

Peripheral blood (plasma, serum, and lymphocyte phenotype) biomarker measurements assist investigators in understanding pathogenesis, treatment monitoring, staging, and progression of a disease [24–26]. IL-6 and its related receptors in interaction with other cytokines play an important role in the pathogenesis and progression of HIV-1 infection. IL-6 affects HIV-1 expression at transcriptional and post-transcriptional stages [27].

The source of high plasma levels of IL-6 and sIL-6R in HIV-1-infected patients appears to be associated with chronic inflammation which may be caused by persistent activation of immune cells due to ongoing production of virus (not on HAART patients) or viral particles by HIV-1 reservoir cells (on HAART patients). Immune activation and inflammatory biomarkers, mainly IL-6, was strongly associated with mortality risk in people living with HIV-1 (PLWH) [28].

The correlation among IL-6 and sIL-6R and especially sgp130 receptors with its complex action is not clearly documented or published for PLWH on HAART. There was **no correlation** among the plasma level of IL-6, sIL-6R, and sgp130 biomarkers in our study. This may be possibly due to **both the agonistic effect of IL-6R on IL-6, antagonistic effect of sgp130 on IL-6/sIL-6R duplex and also suppression of the immune activation by HAART which lead to decline in the secretion of proinflammatory cytokines in HIV-1-infected patients**. In addition, an increased release of proinflammatory cytokines (TNF-α, IL-1ß and IL-6) is seen in HIV-1-infected patients after discontinuation of HAART [18].

The IL-6 signal-transducing receptor gp130 has homeostatic, protective and acute inflammatory functions. Also, the soluble form of gp130(sgp130) acts as a buffer to neutralize the action of IL-6 by binding with the IL6/sIL6R duplex and creating a circulating IL-6-sIL6R-sgp130 complex. In this case the blood levels of the circulating spg130-IL-6-sIL6R complex, in particularly in the chronic stage of disease, [9, 29] may complicate the measurement of free serum levels of these biomarkers which may in turn affect accurate measurement of each biomarker and the correlation among the three biomarkers.

We are not aware of any published literature that examined the correlation among serum levels of IL-6, sIL6R and sgp130 during HIV-1 infection, but there were some reports when looking at other diseases. HIV-1 infection shares some similarities with SARs-CoV-2 infection such as lymphopenia, pro-inflammatory cytokines response, modification of intestinal microbiota, and formation of Neutrophil Extracellular Traps (NETs) involved in pathogenesis [30, 31].

**Ziegler L et al.** investigated the correlation between serum IL-6, sIL-6R, and sgp130 in SARS-CoV-2 infected individuals with the median serum levels of IL-6, sIL-6R, and sgp130 of 39.50 pg/mL, 40.25ng/mL, and 241.31ng/mL respectively. They found that in acute cases of SARS-CoV-2 infected hospitalized patients, there was no correlation among IL-6 and its receptors [32]. The median serum levels of IL-6 and sIL-6R was higher in acute SARS-CoV-2 patients than in the men in our study who were living with chronic HIV-1 infection (34.9 pg/mL vs 1.68 pg/mL for IL-6 and 40.25 ng/mL vs 34.8 ng/mL for sIL-6R respectively). Median serum levels of sgp130 were lower in acute SARS-CoV-2 than in the HIV-1-infected men in our study (241.31 ng/mL vs. 323.00 ng/mL). The data demonstrated that there was no correlation between serum levels of IL-6, sIL-6R and sgp130 in acute SARS-CoV-2 patients and likewise the men living with chronic HIV-1- infection of our study. This data also shows a difference in serum levels of those three biomarkers among the acute and chronic stages of (SARS-CoV-2 and HIV-1) diseases.

**Robak et al.** showed that the serum IL-6R and IL-6 ratio was lower in 66 (57 female and 9 male) rheumatoid arthritis patients (4.114±7.8290 ng/mL) than in healthy individuals (12.976±16.309 ng/mL) [33]. The same pattern was seen in the ratio of sIL-6R/IL6 in our study (23.315±18.999 ng/mL) where HIV-1-infected men had a lower ratio than the HIV-1-uninfected men (28.386±23.577ng/mL). The ratio of IL6R/IL6 was higher in our study which may be due to differences in gender and chronicity of HIV-1 infection. Likewise, there was no correlation between the blood levels of IL-6, and sIL-6R in rheumatoid arthritis, an autoimmune disease.

We assumed that there would be significant correlations among the serum levels of IL-6, sIL-6R, and sgp130 in HIV-1-infected men, but we found no correlation among those three biomarkers in our study, and other studies [32, 33]. This may be possibly due to both the agonistic effect of IL-6R on IL-6, the antagonistic effect of sgp130 on IL-6/sIL-6R and immunosuppression induced by HAART.

One limitation in our study was the lack of acute and untreated HIV-1-infected and women. In addition, due to a shortfall of funding, the quantification of CD126 RFI on CD4^+^ and on CD8^+^ assays were done only on 37 out of 69 HIV-1-infected men and 30 out of 52 HIV-1-uninfected men.

## Conclusion

There was a positive correlation (p<0.05) between plasma levels of HIV-1 RNA and serum levels of IL-6 and sIL-6R in HIV-1-infected men. In Spearman’s correlation, a significant correlation was also noticed between HIV-1 RNA and sgp130, but it was not a linear relationship.

There was no correlation among serum levels of IL-6, sIL-6R, and sgp130 in both HIV-1-infected and uninfected men. Therefore, the action of IL-6 signaling at the cellular level may be more complex than previously thought. It could be possible that the action and circulating blood level of IL-6 and its receptors may be different at each stage of a disease. In addition, measurements of free circulating serum levels of IL-6 and its receptors probably do not reflect the local action of IL-6 and its receptors in a disease. Lack of correlation among serum IL-6, sIL6R, and sgp130 may indicate that each biomarker provides different information about illness progression at different stages of disease.

This study will open a window of opportunity for future investigation of these three biomarkers at different stages of disease progression and at the local level such as synovial fluid.

## Supporting information

S1 Table Pearson’s correlation coefficient of biomarkers for 69 HIV-1 infected men. (PDF)

S2 Table Pearson’s correlation coefficient of biomarkers for 52 HIV-1 uninfected men. (PDF)

S3 Table Spearman correlation coefficient of biomarkers for 69 HIV-1 infected men. (PDF)

S4 Table Spearman correlation coefficient of biomarkers for 52 HIV-1 uninfected men. (PDF)

S5 Table Pearson’s correlation of biomarkers for 38 HIV-1 infected men with IL-6 ≤1.80 pg/mL. (PDF)

S6 Table Pearson’s correlation of biomarkers for 31 HIV-1 infected men with IL-6>1.80 pg/mL. (PDF)

S1 Data (CSV)

## Abbreviations

Abs: Absolute
AIDS: Acquired immunodeficiency syndrome
CD126: Cluster of differentiation of 126
CKD: Chronic Kidney Disease
COPD: Chronic Obstructive Pulmonary Disease
CV: Coefficient of Variation
FITC: Fluorescein Isothiocyanate
HIV: Human Immunodeficiency Virus
IL-6: Interleukin-6
KS: Kaposi’s sarcoma
MACS: Multicenter AIDS Cohort Study
PE: Phycoerythrin
PerCP: Peridinin Chlorophyll Protein
PHA: phytohemagglutinin
RFI: Relative fluorescence Intensity
RT-PCR: reverse transcription–polymerase chain reaction
sgp139: Soluble glycoprotein-130
sIL-6R: Soluble interleukin-6 Receptor
