# Supplementary material for: Relationship among serum levels of IL-6, sIL-6R, s gp130 and CD126 on T-cell in HIV-1 infected and uninfected men participating in the Los Angeles Multi-Center AIDS Cohort Study
Source: PLoS One. 2023 Oct 9;18(10):e0290702. doi: 10.1371/journal.pone.0290702 (PMC10561848; doi:10.1371/journal.pone.0290702)
Supplement: S1 Table — (PDF) [file pone.0290702.s001.pdf]

S1 Table. **Pearson's correlation coefficient of biomarkers for 69 HIV-1-infected men.**

Marker	Abs CD4+	Abs CD8+	RFI of CD38 on CD8+	WBC	LYMPH	AGE	IL-6	sIL-6R	sgp130	RFI of CD126 on CD4+	RFI of CD126 on CD8+
**HIV-1 RNA (Log 10)**	- 0.57097<br><.0001<br>67	0.06173<br>0.6197<br>67	0.72129<br><.0001<br>68	-0.22355<br>0.0690<br>67	-0.18945<br>0.1247<br>67	-0.19122<br>0.1183<br>68	0.39531<br>0.0008<br>68	0.36785<br>0.0020<br>68	0.11773<br>0.3390<br>68	0.04252<br>0.8027<br>37	-0.01593<br>0.9255<br>37
**Abs CD4+**		0.08921<br>0.46494<br>68	-0.50820<br><.0001<br>68	0.47025<br><.0001<br>68	0.32499<br>0.0068<br>68	0.24308<br>0.0458<br>68	-0.12597<br>0.3060<br>68	-0.13144<br>0.2853<br>68	-0.04161<br>0.7362<br>68	-0.03127<br>0.8563<br>36	0.08052<br>0.6406<br>36
**Abs CD8+**			0.01989<br>0.8721<br>68	0.43526<br>0.0002<br>68	0.40940<br>0.0005<br>68	0.02966<br>0.8103<br>68	0.01134<br>0.9269<br>68	0.11450<br>0.3525<br>68	0.32940<br>0.0061<br>68	-0.51968<br>0.0012<br>36	-0.41213<br>0.0125<br>36
**[a]RFI of CD38/CD8+**				-0.25765<br>0.0339<br>68	-0.09732<br>0.4298<br>68	-0.23954<br>0.0474<br>69	0.24358<br>0.0437<br>69	0.41635<br>0.0004<br>69	0.12820<br>0.2938<br>69	-0.09633<br>0.5706<br>37	-0.10161<br>0.5496<br>37
**WBC**					-0.24715<br>0.0422<br>68	0.21832<br>0.0737<br>68	0.04739<br>0.7012<br>68	-0.06319<br>0.6087<br>68	0.21068<br>0.0846<br>68	-0.22534<br>0.1864<br>36	-0.21383<br>0.2105<br>36
**LYMPH**						0.06646<br>0.5902<br>68	-0.21862<br>0.0733<br>68	0.04409<br>0.7211<br>68	0.06196<br>0.6157<br>68	-0.27404<br>0.1058<br>36	-0.11537<br>0.5028<br>36
**AGE**							0.05000<br>0.6833<br>69	-0.12340<br>0.3124<br>69	-0.03186<br>0.7949<br>69	-0.53367<br>0.0007<br>37	-0.51587<br>0.0011<br>37
**IL-6**								0.13174<br>0.2806<br>69	0.02879<br>0.8144<br>69	-0.34342<br>0.0374<br>37	-0.24792<br>0.1390<br>37
**sIL-6R**									0.27677<br>0.0213<br>69	-0.31369<br>0.0587<br>37	-0.13279<br>0.4334<br>37
**sgp130**										-0.38263<br>0.0194<br>37	-0.46230<br>0.0040<br>37
**RFI of CD126/CD4+**											**r: 0.77442**<br>**p: <.0001**<br>**no: 37**

The pair(s) of variables with positive correlation coefficients and p< 0.050 tend to increase together. For the pairs with negative correlation coefficients and p< 0.050, one variable tends to decrease while the other increases. For pairs with p> 0.050, there is no significant relationship between the two variables. [a]RFI: Relative Fluorescence Intensity
