# Supplementary material for: Relationship among serum levels of IL-6, sIL-6R, s gp130 and CD126 on T-cell in HIV-1 infected and uninfected men participating in the Los Angeles Multi-Center AIDS Cohort Study
Source: PLoS One. 2023 Oct 9;18(10):e0290702. doi: 10.1371/journal.pone.0290702 (PMC10561848; doi:10.1371/journal.pone.0290702)
Supplement: S2 Table — (PDF) [file pone.0290702.s002.pdf]

S2 Table. Pearson's correlation coefficient of biomarkers for 52 HIV-1-uninfected men.

Marker	Abs CD8	RFI of CD38 on CD8$^+$	WBC	LYMPH	AGE	IL-6	sIL-6R	sgp130	RFI of CD126 on CD4$^+$	RFI of CD126 on CD8$^+$
**Abs CD4**	0.13989<br>0.3226<br>52	-0.34033<br>0.0136<br>52	0.33684<br>0.0146<br>52	0.32698<br>0.0180<br>52	0.08071<br>0.5695<br>52	-0.11126<br>0.4323<br>52	-0.07020<br>0.6210<br>52	-0.12481<br>0.3829<br>51	-0.23068<br>0.2286<br>29	-0.19243<br>0.3173<br>29
**Abs CD8**		-0.20309<br>0.1487<br>52	0.35482<br>0.0098<br>52	0.11558<br>0.4145<br>52	0.11572<br>0.4140<br>52	0.06495<br>0.6473<br>52	-0.13402<br>0.3435<br>52	-0.01880<br>0.8958<br>51	-0.21419<br>0.2646<br>29	-0.20103<br>0.2957<br>29
**[a]RFI of CD38/CD8$^+$**			-0.43547<br>0.0013<br>52	0.11363<br>0.4225<br>52	-0.09839<br>0.4877<br>52	0.40462<br>0.0029<br>52	0.07103<br>0.6168<br>52	0.13108<br>0.3592<br>51	0.20339<br>0.2900<br>29	0.38360<br>0.0400<br>29
**WBC**				-0.59225<br><.0001<br>52	-0.00859<br>0.9518<br>52	0.29709<br>0.0325<br>52	-0.05591<br>0.6938<br>52	0.01244<br>0.9310<br>51	-0.12476<br>0.5190<br>29	-0.41086<br>0.0268<br>29
**LYMPH**					0.01754<br>0.9018<br>52	-0.31522<br>0.0228<br>52	-0.05660<br>0.6902<br>52	-0.11590<br>0.4180<br>51	-0.04282<br>0.8254<br>29	0.16393<br>0.3955<br>29
**AGE**						0.09191<br>0.5170<br>52	-0.18204<br>0.1965<br>52	0.05739<br>0.6892<br>51	-0.07365<br>0.7042<br>29	-0.12526<br>0.5174<br>29
**IL-6**							0.12894<br>0.3623<br>52	0.14331<br>0.3157<br>51	-0.14072<br>0.4666<br>29	-0.21022<br>0.2737<br>29
**sIL-6R**								0.23017<br>0.1042<br>51	-0.31669<br>0.0942<br>29	-0.04764<br>0.8062<br>29
**sgp130**									0.09416<br>0.6336<br>28	0.05565<br>0.7785<br>28
**RFI of CD126/CD4$^+$**										r 0.70045<br>p <.0001<br>no 29

The pair(s) of variables with positive correlation coefficients and $p < 0.050$ tend to increase together. For the pairs with negative correlation coefficients and $p < 0.050$, one variable tends to decrease while the other increases. For pairs with $p > 0.050$, there is no significant relationship between the two variables. [a]RFI: Relative Fluorescence Intensity.
