# Supplementary material for: Relationship among serum levels of IL-6, sIL-6R, s gp130 and CD126 on T-cell in HIV-1 infected and uninfected men participating in the Los Angeles Multi-Center AIDS Cohort Study
Source: PLoS One. 2023 Oct 9;18(10):e0290702. doi: 10.1371/journal.pone.0290702 (PMC10561848; doi:10.1371/journal.pone.0290702)
Supplement: S3 Table — (PDF) [file pone.0290702.s003.pdf]

S3 Table. Spearman's correlation coefficient of biomarkers for 69 HIV-1-infected men.

Marker	Abs CD4+	Abs CD8+	RFI of CD38 on CD8+	WBC	LYMPH	AGE	IL-6	sIL-6R	sgp130	RFI of CD126 on CD4+	RFI of CD126 on CD8+
HIV-1 RNA (Lin)	-0.60627<br><.0001<br>67	0.08816<br>0.4781<br>67	0.61095<br><.0001<br>68	-0.19717<br>0.1098<br>67	-0.15106<br>0.2224<br>67	-0.19793<br>0.1057<br>68	0.25011<br>0.0397<br>68	0.36414<br>0.0023<br>68	0.30586<br>0.0112<br>68	-0.03904<br>0.8185<br>37	0.01148<br>0.9462<br>37
HIV-1 RNA (Log)	-0.60627<br><.0001<br>67	0.08816<br>0.4781<br>67	0.61095<br><.0001<br>68	-0.19717<br>0.1098<br>67	-0.15106<br>0.2224<br>67	-0.19793<br>0.1057<br>68	0.25011<br>0.0397<br>68	0.36414<br>0.0023<br>68	0.30586<br>0.0112<br>68	-0.03904<br>0.8185<br>37	0.01148<br>0.9462<br>37
Abs CD4+		0.14540<br>0.2368<br>68	-0.58232<br><.0001<br>68	0.38023<br>0.0014<br>68	0.35081<br>0.0034<br>68	0.24245<br>0.0464<br>68	-0.07138<br>0.5630<br>68	-0.04025<br>0.7445<br>68	-0.01098<br>0.9292<br>68	0.08212<br>0.6340<br>36	0.13910<br>0.4185<br>36
Abs CD8+			-0.13034<br>0.2894<br>68	0.39611<br>0.0008<br>68	0.43767<br>0.0002<br>68	0.02470<br>0.8415<br>68	0.10140<br>0.4106<br>68	0.15208<br>0.2157<br>68	0.42796<br>0.0003<br>68	-0.47352<br>0.0035<br>36	-0.39696<br>0.0165<br>36
[a]RFI of CD38/CD8+				-0.29449<br>0.0148<br>68	-0.13634<br>0.2676<br>68	-0.38426<br>0.0011<br>69	0.30238<br>0.0116<br>69	0.22847<br>0.0590<br>69	-0.02121<br>0.8627<br>69	-0.05192<br>0.7602<br>37	-0.05813<br>0.7326<br>37
WBC					-0.27602<br>0.0227<br>68	0.20505<br>0.0935<br>68	0.30022<br>0.0129<br>68	0.04638<br>0.7072<br>68	0.24034<br>0.0484<br>68	-0.13878<br>0.4195<br>36	-0.25519<br>0.1331<br>36
LYMPH						0.03321<br>0.7880<br>68	-0.19853<br>0.1046<br>68	0.03901<br>0.7521<br>68	0.10286<br>0.4039<br>68	-0.24605<br>0.1480<br>36	-0.03833<br>0.8244<br>36
AGE							-0.00684<br>0.9555<br>69	-0.07017<br>0.5667<br>69	0.02590<br>0.8327<br>69	-0.52680<br>0.0008<br>37	-0.52548<br>0.0008<br>37
IL-6								0.19152<br>0.1149<br>69	0.10427<br>0.3939<br>69	-0.33551<br>0.0424<br>37	-0.33691<br>0.0414<br>37
sIL-6R									0.37661<br>0.0014<br>69	-0.39927<br>0.0144<br>37	-0.21590<br>0.1994<br>37
sgp130										-0.39322<br>0.0161<br>37	-0.47117<br>0.0032<br>37
RFI of CD126/CD4+											r 0.79139<br>p <.0001<br>no 37

The pair(s) of variables with positive correlation coefficients and p< 0.050 tend to increase together. For the pairs with negative correlation coefficients and p< 0.050, one variable tends to decrease while the other increases. For pairs with p> 0.050, there is no significant relationship between the two variables. [a]RFI: Relative Fluorescence Intensity.
