# Supplementary material for: Relationship among serum levels of IL-6, sIL-6R, s gp130 and CD126 on T-cell in HIV-1 infected and uninfected men participating in the Los Angeles Multi-Center AIDS Cohort Study
Source: PLoS One. 2023 Oct 9;18(10):e0290702. doi: 10.1371/journal.pone.0290702 (PMC10561848; doi:10.1371/journal.pone.0290702)
Supplement: S4 Table — (PDF) [file pone.0290702.s004.pdf]

S4 Spearman’s correlation coefficient of biomarkers for 52 HIV-1-uninfected men

Markers	Abs CD8$^+$	RFI of CD38/CD8$^+$	WBC	LYMPH	AGE	IL-6	sIL-6R	sgp130	RFI of CD126/CD4$^+$	RFI of CD126/CD8$^+$
**Abs CD4$^+$**	0.0974<br>0.492<br>52	-0.3332<br>0.0158<br>52	0.4869<br>0.0001<br>52	0.0893<br>0.529<br>52	0.0679<br>0.632<br>52	0.0324<br>0.819<br>52	-0.0329<br>0.817<br>52	-0.0259<br>0.857<br>51	-0.1936<br>0.314<br>29	-0.2467<br>0.197<br>29
**Abs CD8$^+$**		-0.2036<br>0.148<br>52	0.2564<br>0.066<br>52	0.1973<br>0.16<br>52	0.0599<br>0.673<br>52	0.0621<br>0.662<br>52	-0.1022<br>0.471<br>52	0.0625<br>0.663<br>51	-0.1557<br>0.42<br>29	-0.1785<br>0.354<br>30
**[a]RFI of CD38/CD8$^+$**			-0.5503<br><0.0001<br>52	0.1975<br>0.16<br>52	-0.2088<br>0.137<br>52	-0.0213<br>0.881<br>52	0.1208<br>0.393<br>52	-0.0932<br>0.516<br>51	0.2219<br>0.247<br>29	0.4239<br>0.022<br>29
**WBC**				-0.6139<br><0.0001<br>52	0.0243<br>0.864<br>52	0.4241<br>0.001<br>52	0.0008<br>0.995<br>52	0.1001<br>0.485<br>51	-0.1945<br>0.869<br>29	-0.4628<br>0.012<br>29
**LYMPH**					-0.0094<br>0.948<br>52	-0.4256<br>0.002<br>52	-0.0397<br>0.779<br>52	-0.1653<br>0.247<br>51	-0.0319<br>0.869<br>29	0.2788<br>0.143<br>29
**AGE**						0.0974<br>0.492<br>52	-0.0906<br>0.523<br>52	0.0884<br>0.537<br>51	-0.1694<br>0.38<br>29	-0.1946<br>0.312<br>29
**IL-6**							0.160<br>0.258<br>52	0.3131<br>0.025<br>51	0.3399<br>0.071<br>29	-0.3135<br>0.098<br>29
**sIL-6R**								0.2529<br>0.073<br>51	-0.2488<br>0.193<br>29	0.0012<br>0.995<br>29
**sgp130**									0.1297<br>0.511<br>28	0.2022<br>0.302<br>28
**RFI of CD126/CD4$^+$**										**r: 0.7082**<br>**p: <0.0001**<br>**no: 29**

The pair(s) of variables with positive correlation coefficients and p< 0.050 tend to increase together. For the pairs with negative correlation coefficients and p< 0.050, one variable tends to decrease while the other increases. For pairs with p> 0.050, there is no significant relationship between the two variables. [a]RFI: Relative Fluorescence Intensity.
