# Supplementary material for: Relationship among serum levels of IL-6, sIL-6R, s gp130 and CD126 on T-cell in HIV-1 infected and uninfected men participating in the Los Angeles Multi-Center AIDS Cohort Study
Source: PLoS One. 2023 Oct 9;18(10):e0290702. doi: 10.1371/journal.pone.0290702 (PMC10561848; doi:10.1371/journal.pone.0290702)
Supplement: S5 Table — (PDF) [file pone.0290702.s005.pdf]

S5 Table. Pearson's correlation coefficient of Biomarkers for 38 HIV-1 infected Men with IL-6≤1.80 pg/mL

Marker	Abs $CD4^+$	Abs $CD8^+$	RFI of CD38 on $CD8^+$	WBC	Lymph	Age	IL-6	sIL6R	sgp130	RFI of CD126 on $CD4^+$	RFI of CD126 on $CD8^+$
**HIV-1 RNA**	-0.304<br>0.0633<br>38	0.134<br>0.424<br>38	0.83<br><0.0001<br>38	-0.167<br>0.316<br>38	0.115<br>0.491<br>38	-0.163<br>0.329<br>38	0.172<br>0.302<br>38	0.688<br><0.0001<br>38	-0.064<br>0.703<br>38	-0.227<br>0.381<br>17	0.0001<br>1.000<br>17
**Abs $CD4^+$**		0.199<br>0.231<br>38	-0.491<br>0.00177<br>38	0.371<br>0.022<br>38	0.31<br>0.058<br>38	0.341<br>0.0364<br>38	0.113<br>0.499<br>38	-0.22<br>0.185<br>38	-0.109<br>0.516<br>38	0.268<br>0.298<br>17	0.259<br>0.315<br>17
**Abs $CD8^+$**			-0.0157<br>0.926<br>38	0.513<br>0.000983<br>38	0.316<br>0.0529<br>38	-0.117<br>0.485<br>38	0.291<br>0.0763<br>38	-0.0133<br>0.937<br>38	0.279<br>0.0894<br>38	-0.291<br>0.257<br>17	-0.0816<br>0.756<br>17
**[a]RFI of CD38 on $CD8^+$**				-0.36<br>0.0266<br>38	0.0804<br>0.631<br>38	-0.306<br>0.0621<br>38	0.11<br>0.509<br>38	0.557<br>0.000277<br>38	-0.0151<br>0.928<br>38	-0.135<br>0.606<br>17	0.0213<br>0.935<br>17
**WBC**					-0.404<br>0.0119<br>38	0.102<br>0.542<br>38	0.341<br>0.0361<br>38	-0.0357<br>0.832<br>38	0.282<br>0.0868<br>38	0.0807<br>0.758<br>17	-0.043<br>0.87<br>17
**Lymph**						0.0831<br>0.62<br>38	-0.0804<br>0.632<br>38	-0.0993<br>0.553<br>38	-0.149<br>0.373<br>38	-0.23<br>0.375<br>17	0.0697<br>0.79<br>17
**Age**							-0.0141<br>0.933<br>38	-0.253<br>0.125<br>38	-0.325<br>0.0463<br>38	-0.202<br>0.437<br>17	-0.288<br>0.262<br>17
**IL-6**								0.0805<br>0.631<br>38	-0.0219<br>0.896<br>38	-0.062<br>0.813<br>17	-0.0147<br>0.955<br>17
**sIL6R**									0.221<br>0.182<br>38	-0.344<br>0.177<br>17	-0.0749<br>0.775<br>17
**sgp130**										-0.388<br>0.124<br>17	-0.486<br>0.0477<br>17
**RFI of CD126 on $CD4^+$**											r 0.613<br>p 0.00885<br>no 17

The pair(s) of variables with positive correlation coefficients and $p < 0.050$ tend to increase together. For the pairs with negative correlation coefficients and $p < 0.050$, one variable tends to decrease while the other increases. For pairs with $p > 0.050$, there is no significant relationship between the two variables. [a]RFI: Relative Fluorescence Intensity.
