# Supplementary material for: Relationship among serum levels of IL-6, sIL-6R, s gp130 and CD126 on T-cell in HIV-1 infected and uninfected men participating in the Los Angeles Multi-Center AIDS Cohort Study
Source: PLoS One. 2023 Oct 9;18(10):e0290702. doi: 10.1371/journal.pone.0290702 (PMC10561848; doi:10.1371/journal.pone.0290702)
Supplement: S6 Table — (PDF) [file pone.0290702.s006.pdf]

S6 Table. Pearson's correlation coefficient of Biomarkers for 31 HIV-1 infected men with IL6>1.80pg/mL

Marker	Abs CD4^{+}	Abs CD8^{+}	RFI of CD38 on CD8^{+}	WBC	Lym	Age	IL-6	sIL6R	sgp130	RFI of CD126 on CD4^{+}	RFI of CD126 on CD8^{+}
**HIV-1 RNA**	-0.251<br>0.189<br>29	-0.025<br>0.898<br>29	0.23<br>0.222<br>30	-0.195<br>0.31<br>29	-0.0954<br>0.623<br>29	-0.157<br>0.409<br>30	0.262<br>0.161<br>30	-0.266<br>0.155<br>30	-0.0848<br>0.656<br>30	0.15<br>0.529<br>20	-0.0381<br>0.873<br>20
**Abs CD4^{+}**		0.0132<br>0.945<br>30	-0.6<br>0.000452<br>30	0.532<br>0.00251<br>30	0.38<br>0.0381<br>30	0.178<br>0.348<br>30	-0.213<br>0.258<br>30	-0.0591<br>0.757<br>30	-0.00339<br>0.986<br>30	-0.165<br>0.5<br>19	-0.0338<br>0.891<br>19
**Abs CD8^{+}**			0.00511<br>0.979<br>30	0.363<br>0.049<br>30	0.544<br>0.00189<br>30	0.172<br>0.364<br>30	-0.0509<br>0.789<br>30	0.329<br>0.0756<br>30	0.371<br>0.0434<br>30	-0.575<br>0.0101<br>19	-0.594<br>0.00738<br>19
**[a]RFI of CD38 on CD8^{+}**				-0.331<br>0.0737<br>30	-0.211<br>0.262<br>30	-0.171<br>0.357<br>31	0.251<br>0.173<br>31	0.189<br>0.307<br>31	0.246<br>0.183<br>31	0.00585<br>0.98<br>20	-0.166<br>0.483<br>20
**WBC**					-0.0395<br>0.836<br>30	0.362<br>0.0494<br>30	-0.102<br>0.592<br>30	-0.167<br>0.378<br>30	0.11<br>0.563<br>30	-0.331<br>0.166<br>19	-0.307<br>0.201<br>19
**Lymph**						0.0466<br>0.807<br>30	-0.231<br>0.219<br>30	0.359<br>0.0516<br>30	0.346<br>0.0612<br>30	-0.317<br>0.186<br>19	-0.258<br>0.287<br>19
**Age**							0.0848<br>0.65<br>31	0.148<br>0.426<br>31	0.34<br>0.0613<br>31	-0.703<br>0.000552<br>20	-0.684<br>0.000876<br>20
**IL-6**								0.271<br>0.141<br>31	-0.0281<br>0.881<br>31	-0.329<br>0.157<br>20	-0.252<br>0.284<br>20
**sIL6R**									0.412<br>0.0212<br>31	-0.494<br>0.0269<br>20	-0.375<br>0.103<br>20
**sgp130**										-0.445<br>0.0495<br>20	-0.491<br>0.028<br>20
**RFI of CD12 on CD4^{+}**											r: 0.898<br>p: <0.0001<br>n: 37

The pair(s) of variables with positive correlation coefficients and p< 0.050 tend to increase together. For the pairs with negative correlation coefficients and p< 0.050, one variable tends to decrease while the other increases. For pairs with p> 0.050, there is no significant relationship between the two variables.

[a]RFI: Relative Fluorescence Intensity.
